# Poster Session I - A57 ARTIFICIAL INTELLIGENCE–ASSISTED ENDOSCOPIC ASSESSMENT OF CROHN’S DISEASE SEVERITY: A SYSTEMATIC REVIEW

**DOI:** 10.1093/jcag/gwaf042.057

**Published:** 2026-02-13

**Authors:** E Hazan, B Nguyen, S Quon, S Singh

**Affiliations:** Gastroenterology and Hepatology, The University of British Columbia Faculty of Medicine, Vancouver, BC, Canada; Medicine, The University of British Columbia, Vancouver, BC, Canada; Medicine, The University of British Columbia, Vancouver, BC, Canada; Gastroenterology and Hepatology, The University of British Columbia Faculty of Medicine, Vancouver, BC, Canada

## Abstract

**Background:**

Endoscopic assessment is integral in guiding the management and monitoring of Crohn’s Disease (CD). While manual scoring systems are widely used, they are subject to significant inter-rater variability. Artificial intelligence (AI) has shown utility in assessing disease activity in ulcerative colitis, yet its role in CD remains underexplored. AI-based tools could improve standardization and efficiency in CD assessment with potential benefits for both clinical practice and research.

**Aims:**

Review the accuracy and potential clinical utility of artificial intelligence-based systems in the endoscopic assessment of CD activity and severity.

**Methods:**

A systematic search of Ovid MEDLINE, Embase, Pubmed, Scopus, and Cochrane database was performed using PRISMA guidelines to identify publications exploring the use of AI-based tools to assess endoscopic disease activity in CD. Studies meeting inclusion criteria were reviewed and statistical measures and other performance metrics were extracted.

**Results:**

Five studies, published between 2021 and 2024, were included. All used convolutional neural networks to analyze still images from capsule or double-balloon endoscopy, comparing model output against expert readings. Each study used a different classification system for mucosal abnormalities, including ulcer severity, inflammatory stricturing, and villous changes. The accuracy, sensitivity, specificity, area-under-the-curve for the models ranged from 62.4-98.4%, 32.4-98.9%, 71.2-99.8%, and 0.565-0.989 respectively.

**Conclusions:**

AI-based tools show considerable promise in the endoscopic assessment of CD severity. However, heterogeneity in their design and performance underscores the need for further validation and standardization prior to adoption in clinical practice.

A57 Table 1: Publications assessing the utility of artificial intelligence in the endoscopic assessment of Crohn’s disease

**Funding Agencies**

None

**Table:** 

Publication	Endoscopy	Classification	Accuracy	Sensitivity	Specificity	AUC
Barash 2021	Capsule	Grade 1 vs Grade 3 ulcer	0.910	0.910	0.913	0.958
		Grade 1 vs Grade 2 ulcer	0.624	0.342	0.712	0.565
		Grade 2 vs Grade 3 ulcer	0.780	0.734	0.912	0.939
Klang 2021	Capsule	All labels	0.794			
		Presence vs Absence of stricture	0.935			0.971
		Ulcerated vs Non-ulcerated stricture	0.789			0.849
Majtner 2021	Capsule	All labels	0.984	0.957	0.998	
Marin-Santos 2022	Capsule	All labels	0.976	0.989	0.963	0.997
Xie 2024	Double-Balloon Enteroscopy	Ulcer presence	0.963	0.948	0.968	0.989
		Non-inflammatory stenosis	0.957	0.951	0.959	0.989
		Inflammatory stenosis	0.967	0.960	0.969	0.992
		Ulcer size (1,2,3)	0.878	0.882, 0.880, 0.859	0.910, 0.883, 0.992	0.952, 0.942, 0.981
		Ulcer surface coverage (1,2,3)	0.873	0.915, 0.821, 0.823	0.876, 0.914, 0.983	0.959, 0.940, 0.980
		Ulcer depth (superficial, deep)	0.852	0.831, 0.868	0.868, 0.831	0.910

AUC: Area under curve

